# The status of causality in biological databases: data resources and data retrieval possibilities to support logical modeling

**DOI:** 10.1093/bib/bbaa390

**Published:** 2020-12-30

**Authors:** Vasundra Touré, Åsmund Flobak, Anna Niarakis, Steven Vercruysse, Martin Kuiper

**Affiliations:** Department of Biology of the Norwegian University of Science and Technology; Residential Oncologist and an Associate Professor; Department of Biology, Univ Evry, University of Paris-Saclay, affiliated with the laboratory GenHotel in Genopole campus, and a delegate at the Lifeware Group, INRIA Saclay; Researcher in computer science and computational biology and focuses on building a bridge between human and computer understanding; systems biology at the Department of Biology of the Norwegian University of Science and Technology

**Keywords:** causal interactions, databases, data representation, interoperability, biological pathway, logical modeling, computational biology

## Abstract

Causal molecular interactions represent key building blocks used in computational modeling, where they facilitate the assembly of regulatory networks. Logical regulatory networks can be used to predict biological and cellular behaviors by system perturbations and *in silico* simulations. Today, broad sets of causal interactions are available in a variety of biological knowledge resources. However, different visions, based on distinct biological interests, have led to the development of multiple ways to describe and annotate causal molecular interactions. It can therefore be challenging to efficiently explore various resources of causal interaction and maintain an overview of recorded contextual information that ensures valid use of the data. This review lists the different types of public resources with causal interactions, the different views on biological processes that they represent, the various data formats they use for data representation and storage, and the data exchange and conversion procedures that are available to extract and download these interactions. This may further raise awareness among the targeted audience, i.e. logical modelers and other scientists interested in molecular causal interactions, but also database managers and curators, about the abundance and variety of causal molecular interaction data, and the variety of tools and approaches to convert them into one interoperable resource.

## Background

Causality is a generic principle describing the effect that one event has on another, but causal associations between a subject and an object can be difficult to ascertain. Correlation could be a sign of causation, but ‘correlation does not imply causation’ [1]. To truly demonstrate causality, observations that unambiguously underpin cause and effect are needed. Sometimes, causality can be determined from an intuitive and Humean understanding [2] (e.g. a ball that is rolling hits another stationary ball, which causes this second ball starting to roll), while in other cases, justifying that A causes B can be hard to demonstrate without thorough evidence (e.g. from a controlled intervention experiment, ample repeatability of the observation, and plausibility).

Systems biology aspires to understand cellular behavior as emerging from a network of molecular mechanisms, and causality constitutes a key concept to clarify how biological entities interact and affect each other. A molecular causal interaction is defined as a biological event where a source entity (i.e. the regulator) affects and changes a target entity (i.e. the regulated entity), in a specific context and with defined biological states of the entities [3]. For example, a protein can affect and regulate the expression of a gene, or it can regulate the activity of another protein or even its own activity (e.g. when a version of a protein stimulates the modification of itself into a different form, or by autocatalysis), or an miRNA can affect its target. These interactions constitute the fundamental pieces of information for network modeling, specifically for discrete computational modeling approaches such as logical modeling (which includes Boolean, i.e. binary values, and multivalued approaches) [4, 5]. Logical modeling employs regulatory graphs which are networks composed of nodes (i.e. biological entities) linked by directed edges that represent information about the effect (i.e. regulation in terms of activation or inhibition) of one or more source entities on a target entity. In addition, the network usually incorporates the logical operators (e.g. AND, OR and NOT) that describe in logical rules how the effects of the different source nodes are integrated as they affect the target node. Each target node has a specific and defined logical formula that describes how its state depends on the combined states of its source nodes. For instance, if two activating source nodes are associated with an ‘AND’ connector, then both of these nodes are required to activate the target. In the case of a logical model, nodes are multivalued, i.e. they can be assigned any possible discrete value, while in a Boolean Network, every node is assigned one of two possible values depending on its state: 0 for absent or inactive, and 1 for present or active. In order to simulate the behavior of a logical model over time, the logical modeling formalism defines that the state of each node in each time step is dictated by the state of its regulators. The simulation’s updating schemes can vary from synchronous (all nodes updated simultaneously) to asynchronous (only one node updated at a time), with many hybrid schemes in between [6]. While the regulatory information embedded in these types of networks may seem basic, they form a reasonable approximation to study biological information processing [7]: logical models are useful when having large networks, limited knowledge about biochemical reactions or limited experimental data which could not fit to other more complex types of models. They can produce valuable biological insights that uncover new biological mechanistic properties [8, 9] (e.g. Collombet *et al.* studied and exposed the mechanism of activation of E2a in lymphoid cell specification [10] and Selvaggio *et al.* were able to assess the importance of tumor microenvironmental signals controlling cancer plasticity [11]) and can provide helpful predictions that benefit biomedical applications [12, 13]. By definition, pure logical models (excluding hybrid logical models) do not use kinetic parameters and can be used to answer qualitative biological questions, such as ‘which phenotype will be favored under given initial conditions’, or ‘what would be the impact of a loss or gain of function mutation to the secretion of a protein’. However, the model building process can be a time-consuming task: each component and its connections need to be carefully described and annotated from reported experimental observations. Up to now, the building of such models was almost exclusively manual, with only a few attempts to automate the construction by using existing prior-knowledge networks complemented with experimental data [14–16]. Nevertheless, the use of existing, curated and well-annotated causal interactions as building blocks of information is expected to help facilitate and accelerate the model building process. Many initiatives in biocuration of causal statements exist today, and an overview of the various resources that this has produced will be of help to the model building community.

We provide a review of publicly available resources and databases that contain ‘explicit’ information (i.e. directed interaction where a source entity regulates a target entity), ‘implicit’ information (i.e. causal relation between entities represented in the process description (PD) type of biological network) or integrated causal information (causal interactions that are incorporated as parts of logical models), as well as tools and pipelines developed to infer causality. Additionally, an overview is provided of the different data formats used to store and download molecular causal interactions, in order to better assess the accessibility of causal interactions and the data interoperability between the resources.

## Molecular causal interaction data resources

**Figure 1:**
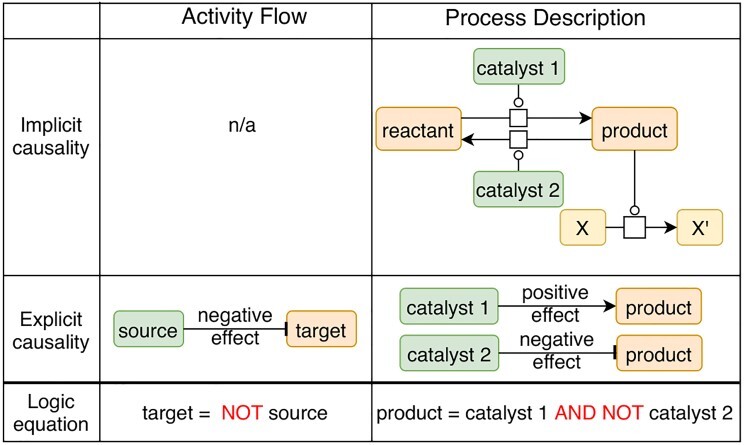
Representation example of explicit and implicit causal interactions in AF and PD. In AF (left panel), causal interactions are evident from the network’s structure: the source entity has an effect on the target entity. In the example, the effect is ‘negative’ [represented with a directed and inhibitory edge (ending in a pipe symbol)]. In PD (right panel), the implicit causality shows a metabolic reaction (state change of an entity) where a reactant entity is consumed to produce a product entity. This reversible reaction is catalyzed by catalyst 1 and catalyst 2. The product acts as a catalyst in a second reaction. Therefore, catalyst 1 has a positive effect on the product since it enables the product to perform its biological function as catalyst. Alternatively, catalyst 2 has a negative effect on the product since it prevents the product to perform that function. The causality can be inferred as follows: both catalyst 1 and catalyst 2 affect the activity of the product entity. The product is in an active state (i.e. the state in which it catalyzes another reaction), and therefore, catalyst 1 activates this particular activity of the product, while catalyst 2 inhibits it. In the case of the AF, the logic equation describes that the target is present or active in the absence of the inhibitor (specified with the operator ‘NOT’). In the case of PD description, the logic equation describes that the product is present or active in the presence of catalyst 1 and in the absence of catalyst 2 (specified with the operators ‘AND’ and ‘NOT’).

Molecular causal interactions can be retrieved from a wide range of resources and databases providing either explicit or implicit causality (see Figure 1 and Table 1), differentiated by different data representation schemes. In the following subsections, we first provide a list of the most prominent databases with explicit causal interactions, i.e. data resources that specifically describe causal interaction with at least a source entity, a target entity and the causal relationship. In addition, we present a list of resources that hold executable logical models, followed by a description of activity flow (AF) pathway resources, as these often also contain useful information for model re-engineering purposes. Next, we will present the resources with implicit causal interactions, such as PD pathway databases where causal interactions must be inferred to be of use for logical modeling. Lastly, we report on computational inference algorithms and tools that were developed to extract causal mechanisms from various biological datasets.

**Table 1:** Summary of the listed explicit and implicit data resources with causal information. The number of causal interactions, models or maps is provided as recorded at the time of writing this manuscript. The `Data provenance' column indicates whether a reference to a manuscript or information source leading to the description of a causal interaction is provided. The `Exports' column indicates the formats in which causal interactions are accessible or in which formats the pathways can be extracted. The `Latest content update' column indicates, to the best of our knowledge, the last year the content of the resource has been modified. The `+' symbol indicates that the database does support a specific characteristic.

	Resource	Data	Data provenance	API	Exports	Latest content update	Species
Causal interactions or AF pathways	CBN [27]	9712 causal interactions	+	+	BEL, SIF	2020	*H. sapiens, R. norvegicus, M. musculus*
	GO-CAM [44]	2956 models	+	+	OWL, GAPB, SIF, JNL	2020	>10 species
	KEGG (AF) [41]	538 pathways, together with PD		+	KGML	2020	>20 species
	SignaLink [22]	89,000 causal interactions	+		SIF	2011	*H. sapiens, D. melanogaster, C. elegans*
	SIGNOR [17, 18]	24,657 causal interactions	+	+	PSI-MITAB2.8	2020	*H. sapiens, R. norvegicus, M. musculus*
	SPIKE [35]	9503 causal interactions	+		SBML, SIF, BioPAX, XML	2012	*H. sapiens*
	Wikipathways [42]	2891 pathways		+	BioPAX, GPML	2020	>20 species
Logical models	Biomodels [37]	18 models	+	+	SBML	2020	*H. sapiens*
	Cell Collective [36]	78 models	+		SBML qual	2020	*H. sapiens, S. cerevisiae, D. melanogaster, etc.*
	GINsim [5]	59 models	+		SBML qual, ZGINML	2020	>10 species
	PyBoolNet [38]	24 models			BNET	2020	*H. sapiens, S. cerevisiae*
PD pathways	ACSN [50]	13 maps	+		SBGN, XML, CellDesigner	2018	*H. sapiens*
	Disease Maps [52]	6 maps	+		SBML, SBGN, BioPAX	2020	*H. sapiens*
	KEGG (PD) [41]	538 pathways, together with AF		+	KGML	2020	>20 species
	PANTHER [47, 48]	177 pathways	+		SBML, BioPAX	2020	142 species
	Reactome [49]	2441 human pathways	+	+	PSI-MITAB2.7, SBML, SBGN, BioPAX	2020	*H. sapiens (curated), 15 more species (inferred)*

### Data resources of causal interactions

Resources of causal interactions describe how a source entity regulates a target entity, and such causal interactions can be directly implemented in a logical model. These resources gather data mainly by manual curation from experimental outcomes or by integrating data from other resources by computational protocols that assess causality between biological entities. In addition to the basic source-regulate-target part, some resources may provide contextual or defining details about the causal interaction, which may help to assess biological conditions in which a causal interaction occurs. In this section, we present three resources providing explicitly causal interactions.

### SIGNOR

The SIGnaling Network Open Resource (SIGNOR [17, 18]), available at https://signor.uniroma2.it/, is currently the most comprehensive biological data resource of manually assessed causal interactions, with more than 23 000 interactions annotated from experiments described in literature. Interactions are defined for three different species: *Homo sapiens*, *Mus musculus* and *Rattus norvegicus.* SIGNOR’s web interface enables the search for causal interactions based on entities of interest, which provides a global vision on the range of connections between the entity of interest with its regulators and targets. This resource mainly focuses on causal interactions between proteins, simple chemicals, complexes, families and phenotypes, where complexes and families are annotated by SIGNOR curators. The annotation of post-translational modifications (PTMs) (i.e. target entities’ modified residues) is recorded, as well as locational information (e.g. cellular component, cell line and tissue). The curated statements can be either computationally accessed through a RESTful service or exported in the PSI-MITAB2.8 [19] format. Note that DISNOR [20], an extension of SIGNOR, uses knowledge from DisGeNET (i.e. a collection of genes and variants associated with human diseases, [21]) to infer disease pathways by linking disease-genes to causal interactions.

### SignaLink

SignaLink2.0 [22], available at http://signalink.org/, constitutes a manually curated resource augmented with automatically inferred causal statements, spanning multiple types of biological interactions (i.e. transcription factor–target gene interactions, miRNA–mRNA interactions and protein–protein interactions). These interactions are assembled to define the pathways of specific biological signaling mechanisms and encompass *H. sapiens*, *Drosophila melanogaster* and *Caenorhabditis elegans*. By integrating different sources, SignaLink associates causal effects between biological components. However, the type of effect (i.e. increase or decrease) of the source upon the target is not always known, presumably due to a lack of information in the original publication or sources. When exporting the whole data in a CSV format, the resource currently (September 2020) consists of more than 89 000 interactions, of which ~74 000 have an ‘unknown’ effect, 14 627 have an ‘inhibition’ effect and 575 have a ‘stimulation’ effect. SignaLink provides exports to several formats, including PSI-MITAB2.7 [23], BioPAX level 3 (i.e. standard format for exchanging pathway information [24]) and SBML (i.e. the Systems Biology Markup Language, a standard format commonly used for storing biological models [25, 26]).

### Causal Biological Networks

The Causal Biological Networks (CBNs) database [27], available at http://causalbionet.com/, is a manually curated signaling pathways database, currently focused on biological processes in pulmonary and vascular systems [28–33]. This database contains both causal and non-causal interactions and combines these interactions into 46 modular networks leading to specific phenotypes for each taxon (i.e. *H. sapiens*, *M. musculus* and *R. norvegicus*). CBN not only defines interactions between physical entities such as genes, RNAs, proteins, protein fusions, protein complexes and small molecules but also 9712 causal interactions between activities including biological processes, phenotypes and pathologies, as well as interactions between entities and activities. The data can be exported in a JSON Graph File (JGF, https://jsongraphformat.info/) and the Simple Interaction File (SIF). In addition, the CBN-BEL converter (https://github.com/pybel/cbn-bel) enables the export of these networks in the Biological Expression Language (BEL, [34]).

The above resources constitute the most comprehensive and popular ones in the domain but several other databases exist (e.g. SPIKE [35]), but these are often smaller, lack annotation detail or have a small user base. However, these causal interaction resources are not the only source of causal interaction information, as described in the next subsections.

### Data resources of logical models

Another class of resources delivers assembled logical models, which are the collections of causal interactions connected by logical rules. These models are commonly contextualized to describe specific biological mechanisms or phenotypes and are executable in logical simulation frameworks. These resources include Cell Collective [36], Biomodels [37], the GINsim repository [5] and the PyBoolNet repository [38]. They constitute collaborative platforms for building and/or sharing manually curated models exportable in the SBML qual format [39], which is an extension of SBML that supports qualitative modeling. A user can therefore either download a model of interest to use the whole or parts of it in their modeling work, or make use of platform tools to do simulations (i.e. with Cell Collective, GINsim and PyBoolNet). Logical model resources constitute valuable knowledge of ready-to-use integrated pieces of causal information.

### Data resources of AF pathways (explicit causality)

Resources of AF pathways describe the collections of causal relations between biological entities or specific activities of biological entities, connected together. Unlike causal interaction resources, where only one interaction at the time is delineated, AF pathways connect several causal interactions to depict specific biological mechanisms. This type of data representation has been standardized in the System Biology Graphical Notation (SBGN [40]) AF language. These interactions are regulatory and have a specific biological effect (e.g. ‘source’ negatively regulates ‘target’, see Figure 1). Several pathway databases represent signaling events in an AF form. For instance, the Kyoto Encyclopedia of Genes and Genomes (KEGG, [41]) is a curated database with several categories focusing on the understanding of functions of biological systems (e.g. the cell, the organism and the ecosystem), among others from information at the molecular level obtained through genome sequencing and high-throughput experimental technologies. A subset of KEGG pathways describes signaling events in the AF form. Similarly, WikiPathways [42, 43] is a growing, collaborative platform for the curation, dissemination, visualization and analysis of biological pathways, focusing on genes, proteins and metabolites. Each pathway has a wiki page that contains a detailed description, visualization and the possibility to download the pathway in different formats. Finally, the Gene Ontology Causal Activity Models (GO-CAM, [44]) consists of pathways annotated (manually or computationally) with the Gene Ontology (GO) terminology [45] (i.e. with GO terms describing biological processes, molecular functions and cellular components). Causality between entities in a GO-CAM model is defined via building blocks of the form: ‘a molecular function, enabled by a gene product, regulates a molecular function, enabled by the same or another gene product’. Therefore, causality is defined in an activity-centric way in these models, and gene products are the entities that execute these activities. GO-CAMs also enable the depiction of causal regulation between biological processes, and between a molecular function and a biological process. The models are created via the web-based collaborative editor Noctua, http://noctua.berkeleybop.org.

However, not all pathway databases represent information for causal interactions in an explicit format (i.e. source entity regulating a target entity). Several pathway databases adopt the ‘PD’ form to express biological mechanisms which may carry implicit causality as described in the following section.

### Data resources of PD pathways that carry implicit causality

Pathway databases are commonly built to provide a comprehensive representation of known biological mechanisms, which are portrayed as biochemical reactions. The reactions usually represent the transformation of molecules (i.e. reactants) that are consumed to produce other molecules (i.e. products), catalyzed by enzymes. These biochemical reactions are visualized with the SBGN PD standard language [46]. SBGN PD pathways commonly refer to metabolic and signaling representations of biological events (i.e. networks and interactions) where the mechanistic details are preserved: entities go through physical or locational state changes, sometimes because of the action of a catalyst or regulator (e.g. A catalyzes the reaction transforming B1 in B2, where B2 is a phosphorylated form of B1). While these networks can be used for Ordinary Differential Equation (ODE) modeling, the causality associated with these ODE relationships makes them interesting for logical modeling as well. However, the causal aspect between the entities of these types of networks is buried in the PD representation, which is why we name them ‘implicit causalities’. In this case, an inference step is required to make the causal relation between two entities explicit, based on the knowledge provided by the reaction as described in Figure 1.

Popular pathway databases with signaling and gene regulatory information include the already mentioned KEGG (its PD pathways), Protein ANalysis THrough Evolutionary Relationships (PANTHER) [47, 48], Reactome [49], Atlas of Cancer Signaling Network (ACSN) [50] and Disease Maps [51, 52]. These manually curated resources are used by a wide range of scientists for understanding the mechanistic aspects of regulatory pathways and to interpret the outcome of experimental results. The PANTHER resource contains 177 pathways with relationships between interacting biological entities, constructed with modeling tools such as CellDesigner [53]. The Reactome pathway database is a resource of biological mechanisms, analysis algorithms and predictive computational models. ACSN is a repository of biological networks focused on the representation and analysis of cancer regulatory networks (i.e. molecular processes found in cancer cells and tumor microenvironment). Disease Maps represent a growing community effort that builds a collection of biological networks representing mechanisms affecting a wide range of diseases (e.g. COVID-19 Disease Map [54], Parkinson’s Disease Map [55], AsthmaMap [56] and RA-map [57, 58]). The Disease Maps are archived in the Minerva platform [59] and involve clinicians and domain experts to validate the annotated pathways. In addition to these resources, several more narrowly focused yet valuable efforts on specific diseases of processes have been published and their results are made available as pathway maps describing specific normal or diseased biological conditions [60–66].

The extraction of causal interactions from these pathway databases needs a conversion of the data structure in order to explicitly represent causality for logical modeling. Several studies have been performed to identify the patterns of various biological interactions that would allow their translation into causal interactions between biological entities. For instance, Vogt *et al.* [67] proposed rules to translate maps from the SBGN PD language to SBGN AF to obtain smaller and more manageable maps, which has been implemented in the SBGN-ED tool [68]. Recently, Aghamiri *et al.* developed CaSQ, [15] a tool to automatically infer executable Boolean models from molecular interaction maps represented in the CellDesigner [53] format. The researchers proposed a framework of graph conversion for PD representations including SBGN schemes, concerning various biological scenarios such as complex formation, phosphorylation, ligand-receptor interaction, etc., with simultaneous inference of logical formulae describing regulation. The logical rules are inferred based on the topology and semantics already encoded in the original maps, resulting in Boolean models with an AF-like layout. The tool is able to process large and complex maps and produce Boolean models in the standard output format SBML qual that is directly executable using popular modeling tools. References, annotations and layout of the CellDesigner molecular maps are retained in the obtained model, facilitating visual inspection, interoperability and reusability of the content. Moreover, the AF structure of the CaSQ-inferred Boolean models can be obtained in the SIF format. Both Vogt *et al.* and Aghamiri *et al.* base their graph conversion rules on the topology of PD pathways (i.e. each reaction will have a specific translation defined) and the semantics already encoded in the glyphs and arcs used for constructing these pathways (i.e. inhibition arcs, complex formation glyphs, etc.).

### Integrated resources to access causal interactions and build models

While data resources presented above have their own curation efforts and allow the download of causal interactions through different data formats to access the curated knowledge, several databases focus less on curation but integrate information from other resources to combine knowledge describing causal interactions into a single platform. These resources usually provide powerful querying interfaces, via command line, web services, graphical interfaces or scripting, which facilitates data retrieval for a particular study (see Supplementary File S1 available online at https://academic.oup.com/bib). OmniPath is a comprehensive integrated resource that combines a vast range of curated and computed signaling resources (full list available at http://omnipathdb.org/), to offer a single endpoint for querying data [69, 70]. They can be accessed and analyzed through pypath (http://saezlab.github.io/pypath), a Python module enabling the construction of models from the multiple supported resources. BioGateway [71] is a semantic knowledge base with curated information from a variety of Elixir core data resources [72] and recently augmented with a knowledge graph containing an exhaustive set of regulatory interactions between transcription factors and target genes (98 768 interactions, albeit only a minority with regulatory type), which can be queried using the SPARQL query language. The BioGateway app [73] provides access from the Cytoscape network editor and allows the export of data in all the formats supported by Cytoscape, including the SIF format. NDEx [74] is an online network data exchange platform that provides a ‘commons’ of biological networks. The resource aims to be a collaborative platform where scientists can deposit and share their networks, as well as use resources from others with multiple application possibilities such as visualization and analysis of the networks in Cytoscape. Likewise, the Pathway Commons resource incorporates knowledge from 22 pathway and interaction databases [75] and enables to export data in BioPAX and SIF, among other formats. PathMe aligns and integrates pathways from KEGG, Reactome and WikiPathways in BEL [76], and the greater Bio2BEL [77] ecosystem which it is part of integrates more than 50 biological databases that can be exported to the different formats supported by PyBEL [34].

## Causal inference in software and pipelines

In addition to resources providing causal molecular interaction information, a broad set of pipelines have been produced to infer causality from various types of biological datasets. This inferred knowledge is generally coupled with prior knowledge extracted from literature. We provide a brief overview of these tools. The INDRA-IPM modeling web interface enables the assessment of causality by translating biological mechanisms from natural language processing [78] and enabling the export of the models in various standard formats (e.g. SBML [25] and SBGN [40]). The CAusal Reasoning for Network identification using Integer VALue programming (CARNIVAL [79]) is a pipeline that integrates gene expression data to identify upstream regulatory signaling pathways. CARNIVAL implements a reverse engineering process and uses a prior knowledge network obtained from OmniPath [69, 70]. Similarly, CausalR [80] is an R package that extracts causality from genome expression datasets. CoRegNet [81] is an R package that infers co-regulatory networks of transcription factors and target genes by analyzing transcriptomics datasets and estimating activities of transcription factors. Whistle [82] implements the Reverse Causal Reasoning algorithm to discover upstream causal regulators from transcription profiles. It uses prior knowledge described in the BEL format to identify possible molecular mechanisms that explain the gene expression data. CausalPath [83] infers causal interactions from prior knowledge resources (i.e. Pathway databases) combined with proteomics cell lines profiles. This technique enables the automatic contextualization of causal interactions for diseases in which they are experimentally observed. CausalPath uses mainly the Pathway Commons data to assess causality [75]. CANDis is a web server that explores possible causal relations between diseases, drugs and drug targets [84]. Bayesian methods have been used in several studies to infer causality: SAGA [85] infers transcriptional regulatory relationships and ARACNE [86] enables the reconstruction of mammalian transcriptional regulatory networks using both biological data (i.e. microarray datasets) and mathematical methods (i.e. Relevance Networks and Bayesian Networks algorithms). Martin *et al.* [87] developed a scoring method applied in their study to the biomedical field (e.g. impact of disease, drug treatment and environmental agents on humans), the Network Perturbation Amplitude (NPA). When combined with high-throughput data and prior knowledge, the NPA algorithm identifies change of activities in targeted biological processes and thereby helps to better understand biological mechanisms leading to diseases. TETRAD (http://www.phil.cmu.edu/tetrad) is a software implementing algorithms, such as Fast Greedy Equivalence Search and Greedy Fast Causal Inference, to enable causal inference. The application has been applied to projects inferring causality for biological and clinical molecular interactions [88]. Finally, Causaly (www.causaly.com) is a commercial interface that uses artificial intelligence, machine learning and text mining to infer evidence and causality from biomedical data. A variety of data sources, including Biomedical Literature, Clinicaltrials.gov and several side-effect databases are machine-read and integrated in the Causaly Knowledge Graph. All evidence is represented as a cause-effect network operating in a graph database and can be queried and explored through a defined REST API to extract relevant causal interactions or even infer logical rules in a model.

## Data exchange formats

Formats have been developed from multiple perspectives, each supporting the expression of causal interactions with different aspects that answer specific use cases. They enable the retrieval of causal interactions through the download of files for different usage purposes. These formats can range from the simplest form with two entities and the regulation sign (e.g. SIF) and more complex representations with the storage of additional metadata [e.g. the BEL, GO-CAM using the Web Ontology Language (OWL), and PSI-MITAB]. The following section aims at providing a short description of the most prominent formats (detailed description can be found through the links provided in Supplementary File S2, available online at https://academic.oup.com/bib), the type of metadata these formats can handle and the list of databases providing causal interactions in these formats.

### The Simple Interaction File

The SIF is a space- or tab-delimited format composed of three elements per line: the source node, the interaction type and the target node. Each line of data corresponds to a single interaction. SIF constitutes the most simple representation of causal interactions and can be used in logical modeling for providing a model topology (the regulatory graph), but it will not provide full information about the logical rules; information about logical operators is also needed and may need additional efforts in curation or other approaches to infer the complete configuration of these rules. In addition, there is no standard agreement for annotating these interactions in SIF. It is nevertheless common to represent the source and target node with a name (e.g. MYC, for HGNC-named genes [89]) or an identifier (e.g. P01106, for a UniProt entry [90]) of the matching biological entity. The interaction type is annotated with terms such as ‘activates’/‘inhibits’, ‘increases’/‘decreases’, ‘up-regulates’/‘down-regulates’ or even symbols such as ‘→’/‘--|’ to represent the causal effect of the interaction. Still, the data annotation should be consistent throughout each individual data resource. As the SIF file contains only the most basic yet important information about an entity-based causal interaction (i.e. no contextual details are stored), it can be seen as a format for data users once they have filtered for data of interest to their study (e.g. selected for appropriate biological context) from original knowledge sources. A SIF file can be easily used in visualization tools for displaying and analyzing networks (e.g. Cytoscape [91]). Several databases allow export into a SIF format, such as Cell Collective [36], OmniPath [69, 70] and CBN [27].

### The PSI-MITAB2.8, a tab-delimited standardized format

The Molecular Interactions community from the Human Proteome Organization Proteomics Standards Initiative recently invested efforts in the representation of causal details in Molecular Interactions (PSI-MI) [92]. Initially focused on the representation of molecular interactions, the directionality and effects were not supported by the PSI-MITAB2.7, a tab-delimited format used for the curation of molecular interactions. A recent upgrade of the PSI-MITAB format included the support for both directionality and effects of molecular interactions, thus supporting the representation of entity-based causality. This extension called ‘CausalTAB’ [19] or ‘PSI-MITAB2.8’ extends the PSI-MITAB2.7 with four features to support causal information: the ‘Biological effect of interactor A’ and ‘Biological effect of interactor B’, informing about the activated molecular function (i.e. an entity’s activity) in the causal interaction, the ‘Causal regulatory mechanism’ reporting the biological mechanism underlying a causal interaction, and the ‘Causal statement’ informing about the effect of the causal interaction (e.g. up-regulation or down-regulation). In addition, the MI controlled vocabulary has been extended with a ‘causal interaction’ branch to incorporate terminology to define regulatory terms [19]. PSI-MITAB2.8 is a standard format supported by the SIGNOR database, version 1.4 of the PSICQUIC web service [23], and it is expected that other resources will also enable the storage and retrieval of causality in this format.

### The BEL, a triplet-oriented format

The BEL (https://bel.bio/) is developed to express causative or correlated relations observed in a specific biological context using BEL statements [34]. A BEL statement is a semantic triplet of information composed of a subject (the regulator), a predicate (e.g. a causal interaction such as increase, decrease and variations of these) and an object (the target). For the subject and the object, a reference to the biological entities using namespaces (controlled vocabularies specifying their origin) is given and their related biological conditions are annotated with BEL functions (e.g. protein modification, binding, kinase activity or epigenetic modifications [93]). Thus, BEL enables the representation of both activity- and process-based causal interactions. In addition, contextual annotations (e.g. evidence, experimental context and cell line) can be associated with a BEL statement thanks to the use of BEL annotations. The main advantage of a BEL statement lies in the fact that it presents a relatively simple and flexible syntax (e.g. PD-like and AF-like statements can be mixed) meaningful both to humans and computers. The Python package PyBEL has been developed to enable the parsing and validation of BEL scripts, and conversion to other formats [34]. Several resources provide export of causal interactions in the BEL format, which are listed at: https://cthoyt.com/2020/04/30/public-bel-content.html).

### The GO-CAM, an OWL/RDF format

The GO-CAM [44] is a formalism developed for structuring biological processes by defining triplets of information (subject, predicate and object) using the OWL representation [94]. GO-CAM is based on the GO terminology and uses the Relation Ontology (RO) terms for the annotation of causal interactions [95]. The RO contains a branch called ‘causally related to’ that groups terms for representing causal relations between material entities, between processes and between both a material entity and a process. Still, GO-CAM is mainly based on an activity-level or reaction-level representation of causality rather than an entity-level based causality (even though the latter can be inferred). In this activity-based view, causality is represented as follows: a specific molecular function (e.g. a phosphorylation) that is enabled by an entity regulates a specific molecular function of another entity. GO-CAM is robust in terms of computational aspects: OWL is a powerful representation that structures knowledge in a way that allows for reasoning and offers the potential for real-time consistency checking during a curation process. But its understanding by humans is cumbersome at best. To address this and to help curators, the Noctua annotation platform is being developed (http://noctua.geneontology.org/), providing a graphical user interface for assembling, editing and interpretation of GO-CAM models.

### The Systems Biology Markup Language for qualitative models (SBML qual), an XML-based standard format to describe regulation using the logical formalism

SBML qual [39] is an XML-based standard developed by the Consortium for Logical Models and Tools (CoLoMoTo, http://www.colomoto.org/) and adopted by the SBML community, which is part of the COMBINE initiative [96]. It constitutes a more computational simulation-oriented format that is primarily meant to support exports of logical models. The mathematical framework encoded in this format facilitates the simulation of the annotated models. Consequently, SBML qual is suited for storing the collections of entity-based causal interactions and this provides ready-to-use models for modeling software that support this standard (e.g. CellNOpt [97], GINsim [5], BoolNet [98],VisiBool [99] and Cell Collective [36]).

**Figure 2:**
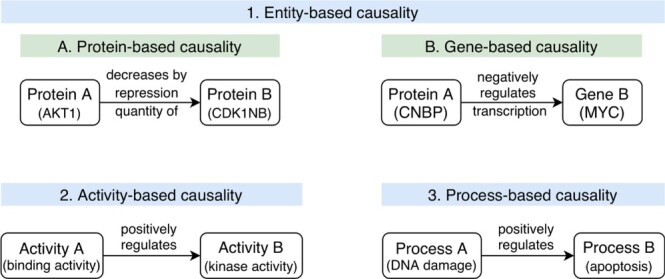
Different representations of causal interactions. (**1A**) Protein-based causal interaction where both entities are gene products: Protein A (AKT1) represses the activity of Protein B (CDK1NB) by decreasing the quantity of protein by repression (meaning that this is most likely an indirect regulation with an intermediate entity, i.e. the gene that enables the production of Protein B); (**1B**) Gene-based causal interaction where Protein A negatively regulates the transcription of Gene B; (**2**) Activity-based causality where an Activity A positively regulates an Activity B. Usually, the entities that perform these activities are known and annotated; (**3**) Process-based causality where a biologicxal Process A (DNA damage) positively regulates a biological Process B (apoptosis).

## Discussion

### The different views on causal interactions

Over time, scientists have adopted different visions to describe causality in causal interaction data resources, based on their biological interest and subsequent data analysis purposes (see Figure 2). Data resources with entity-centric description of causal interactions are predominant. In these representations, the focus is commonly placed on proteins and transcripts as actors of the causal interaction (protein-centric). The protein-centric causal statements may not report in detail gene regulatory events (see Figure 2, Example 1.1). In SIGNOR, for instance, the causal relation between a transcription factor and a target gene is assessed by the transcription factor (source) indirectly regulating the gene product. In a gene-centric view, the mechanistic details of gene regulation would be represented and the gene itself would be represented as the target entity (see Figure 2, Example 1.2). The gene does not have an activity per se, but the binding of a transcription factor to a regulatory sequence of the gene induces the process of transcription and thus the expression of the RNA. The gene-centric view allows context-dependent modifications of the gene to be represented (e.g. histone or DNA methylation) and the exact biological mechanisms could be unambiguously described, but it would make the modeling process more complex because more entities would be introduced. Other resources, such as GO-CAM, focus not on the genes, but on the biological activities of gene products, and represent these activities as being the source and target ‘entities’ of the causal interaction (see Figure 2, Example 2), therefore called ‘activity-based causality’. These activities are usually linked to a gene product. Finally, process-based causality additionally incorporates one or two biological processes as entities, usually as a target entity to describe a phenotypic outcome (see Figure 2, Example 3).

### Interoperability between data formats

#### Annotation differences between formats

Data interoperability depends on the ability of a certain data format to meet the demands of different computer systems or tools, to exchange, comprehend and perform meaningful processing of this data. After reviewing the different formats for storing causal interactions, it is noticeable that they all share a common core of information: two interacting entities (source and target, which can be physical entities or activities) and the regulation type (effect) of this interaction. This set of information is covered in the SIF format, commonly used as input format by network modelers in computational modeling frameworks that connect interactions together. However, the interesting aspects reside in their differences. These differences are influenced on one side by the data annotators, who format the knowledge that is accessible from primary data (e.g. literature and experiments), and on the other side by the end users, who need specific knowledge defining the context in which a causal interaction is applicable. The annotations and contextual differences are listed in Table 2. For instance, BEL and PSI-MITAB2.8 explicitly mention and structure the PTMs (e.g. phosphorylation and acetylation) and cellular locations of the biological entities. Notice that such defining detail or ‘context’ enables end users to build more accurate models, as it informs them on the validity of chaining together several individual causal interactions, whereby the target entity from one causal interaction is used as the source entity for, the next one, in the assembled model. This flexible reuse of a causal statement needs information about the ‘state’ of a protein and, hence, the PTM status, since the PTM to a large extent determines (one of) a protein’s active versus inactive states. Also, both of its original cellular compartments may be informative for assessing the validity of the assembled logical model or for fine-tuning it to better match available experimental data. Notice that the description of PTMs can also vary between formats, meaning that there is no agreed consensus to represent this information. An effort to address this issue has been proposed by Danos *et al.* [100], in the context of rule-based modeling. Furthermore, BEL, GO-CAM and PSI-MITAB2.8 allow to depict with precision the biological activities of the entities involved, using GO terms. BEL in addition supports representing some of these activities using an internally defined vocabulary of a dozen high-level, shorthand terms (e.g. ‘kin’ for kinase activity), which PyBEL can map to GO terms (https://pybel.readthedocs.io/en/latest/_modules/pybel/language.html).

**Table 2:** Comparison of annotations in different formats for causal molecular interactions. Data and metadata types, described in the MI2CAST guidelines, that can currently be annotated and stored in each format are listed with the ontologies and controlled vocabularies used. If there is no specification about ontologies and controlled vocabularies to use, the `+' sign is given, meaning that the format stores explicitly this type of data or metadata. Table inspired from [3].

	SIF	SBML qual	PSI-MITAB2.8	GO-CAM	BEL
Source entity	+	+	UniProtKB, RefSeq, ChEBI, EMBL/DDBJ/GenBank, Entrez Gene, Ensembl, EnsemblGenome	UniProtKB, Model Organism Database (MOD) gene identifier, Protein Ontology	HGNC, ChEBI, RefSeq, Entrez Gene, etc.
Interaction effect	+	+	PSI-MI	RO	BEL term: increases, decreases, etc.
Target entity	+	+	UniProtKB, RefSeq, ChEBI, EMBL/DDBJ/GenBank, EntrezGene, Ensembl, EnsemblGenome	UniProtKB, Model Organism Database (MOD) gene identifier, Protein Ontology	HGNC, ChEBI, RefSeq, etc.
Reference			PMID	PMID or MOD reference	PMID
Evidence			PSI-MI	ECO	ECO
Experimental setup					ECO
Biological mechanism			PSI-MI	combination of biological activity and relationship	GO:BP
Biological activity			GO:MF	GO:MF	BEL term: act((prefix:id), ma(prefix:id)), etc.
Biological type			PSI-MI		BEL term: g(), r(), etc.See documentation.
Biological modification			PSI-MOD	Protein Ontology	BEL term: p(prefix:identifier, pmod(prefix:identifier)), etc.
Taxon entity			NCBI Taxonomy	NCBI Taxonomy	NCBI Taxonomy
Taxon interaction			NCBI Taxonomy		NCBI Taxonomy
Tissue type				Uberon (animals), Plant Ontology, Fungal Anatomy Ontology, MOD-specific ontologies	BRENDA Tissue Ontology
Cell type/Cell line				Cell Ontology, MOD-specific ontologies	BRENDA Tissue Ontology, Cell Ontology, Cell Type Ontology
Cellular compartment		+		GO:CC	GO:CC

The absence of common format guidelines leads to possible data interoperability issues [101], as it can also be observed in Table 2 where annotations types and ontologies and controlled vocabularies used may differ. The building of these different data resources often remains a niche activity, making it arduous for data users to build data integration protocols that are able to cope with the diversity of public data. In addition, their subsequent data analysis approaches are limited by the dataset with the least expressive value. And even if the level of annotation detail is the same, the use of a specific ontology or controlled vocabulary can vary across resources. This results in non-compatible data sets and it would demand rigorous mapping services between these sets to meet the needs of the data users. For instance, the same biological entity may be annotated and referred to different identifiers: BEL and PSI-MITAB2.8 commonly use UniProt IDs [90] for proteins but BEL also enables the use of HGNC IDs [89] for the annotation of gene products, and whereby the context in which an identifier is used clarifies whether the identifier refers to a gene or to a specific type of gene product (e.g. having kinase activity implies that the referred biological entity is a protein). For these reasons, data aggregation between databases can be a challenging task, or at least require additional data processing tasks. This is further compounded by the fact that comparing causal statements between resources may lead to seemingly conflicting causal interactions because of the heterogeneous contextual information (and the extent to which it is supported in different formats), thereby leaving the data analyst with the task to solve possible ambiguities.

#### Causal interaction formats for logical modeling

In logical modeling, the focus is put on entities, representing ‘nodes’ in a regulatory network. On the one hand, SIF, PSI-MITAB2.8 and SBML qual lean toward an entity-based representation of causality, which makes these formats more amenable for combining causal regulatory interactions into logical statements as inputs for logical modeling. On the other hand, GO-CAM and BEL allow the representation of causality from an activity-based view for the biological entities, and a process-based view for biological reactions, meaning that the focus is not put on biological entities per se but rather on functions or actions that they can perform. In this case, the use of statements for logical modeling may require a minor post-processing of the data to extract entities’ information, as logical models do not explicitly focus on activities or processes, or at least they do not represent that level of details. It should be noted though that BEL in principle supports both views and that information about the biological activities is also supported in PSI-MITAB2.8 as a defining detail.

Different formats are built to serve different purposes and needs. For storing any type of contextualized causal interactions, the PSI-MITAB2.8, BEL and GO-CAM formats seem to be well suited as they can handle fine-grained details describing a causal interaction. These formats facilitate the pre-processing work for the modelers who can extract relevant causal interactions through filtering processes: a defined context allows to better assess whether a causal interaction is useful or valid for the specific biological study. The SIF and SBML qual formats are better suited for handling a collection of causal interactions that together form a model for a specific context or case study. SBML qual can already store logical formulae describing the causal regulations, as well as annotations and references that support the causal statement. These files are mainly used as inputs of modeling or simulation tools (e.g. GINsim and Cell Collective), and after running the simulations, they are also used to further analyze the outcomes of causal interaction networks for a given biological situation. In theory, PSI-MITAB2.8, BEL and GO-CAM can also be used as input files in modeling tools when the interactions of interest have been selected. These statements of course only apply when a particular tool supports the import of these formats. It should be noted that for instance, in the case of PSI-MITAB2.8, additional steps are required to correctly assess information about complexes (i.e. collections of entities acting together), because this format stores a protein complex as a list of several binary interactions. These binary interactions need to be combined when building a model, in order to correctly assess the cases in which an entity is acting in combination with other entities (i.e. AND logic formulae will be added between the components of a complex). This exemplifies that different formats have different trade-offs. Managing biomolecular process information necessitates representing entities of diverse compositions, and in diverse and highly specific states (even after translating their diverse interactions into causal regulations). Tabular data (such as PSI-MITAB2.8) are a convenient input format for data analysis algorithms, but due to the format’s inherent limitations for representing this kind of information variability (as shown for protein complexes), it can still require an extra preprocessing step. A non-tabular format (such as BEL) can support more of this information variability but then again may equally require a dedicated preprocessing step before serving as input for modeling algorithms.

#### Standard guidelines to improve interoperability

The utility of these various sources of causal interaction data holds room for improvement. Molecular causal interactions as such may be improved if producers adhere to the recently proposed Minimum Information about a Molecular Interaction Causal Statement (MI2CAST, [3]) guidelines, which guides curators to add a wide range of annotation details (see Table 2) that will significantly increase the utility of the information for logical modelers. MI2CAST has been proposed through the collaboration of scientific communities involved in causal representation (e.g. NTNU, IMEx consortium, GREEKC consortium (http://greekc.org/) and Swiss Bioinformatics Institute), which organized discussions between data curators, data providers and data users wishing to improve and standardize the curation of causal molecular interactions. The establishment of these guidelines encourages the communities to update data formats (PSI-MITAB2.8, GO-CAM and BEL) and curation protocols to comply with MI2CAST and offer better interoperability between resources supporting these formats.

Beyond MI2CAST, there is a clear need for additional curation detail, in particular for context details that provide input to the logical rules. Source nodes that are members of a complex, or need to act additively to cause an effect, may already be equipped with annotation details specifying in which context AND operators need to be used. In addition, causal interactions describe mainly the effect of a source entity on a target entity. However, when building a model that combines causal interactions, a target entity usually has more than one source entity (regulator). The way regulators affect the target entity is defined through the use of logical operators (e.g. AND, OR and NOT) that together define when the target entity is active or not. These logical rules are not necessarily described in existing causal interactions resources or formats (except logical model resources and in SBML qual) and need to be assessed by modelers either through computer algorithms or manual curation when building their models. This issue should be discussed within the relevant communities (i.e. MI2CAST, CoLoMoTo, Disease Maps and COMBINE) [102] to decide how context-specific information that is essential for specifying the logical rules can be recorded during the curation process, and how this can be added to the MI2CAST curation guidelines.

To facilitate the validation of logical models and improve their quality through the use of annotated causal interactions, the CoLoMoTo/SysMod communities have recently put in place the CALM (curation and annotation standards for logical models in biology) roadmap to propose the adoption of good curation and annotation practices when building logical models. It suggests to include integrated pipelines to facilitate data reproducibility, to follow minimum requirement guidelines and use standards (i.e. MIRIAM identifiers, unique protein and gene identifiers, RDFs, SBML qual format, etc.) for increasing data interoperability, to put effort in the development of automatic annotation tools for reducing the gaps between curation and model annotation, and to systematically use a common repository (i.e. Cell Collective [36], Biomodels [37] and the GINsim model repository [5]) for sharing the produced models.

For the interoperable use of AF and PD graph information, conversion or mapping tools that translate between these formats are needed to seamlessly translate between any two formats, to further derive value from all the individual lines of model building and analysis.

**Figure 3:**
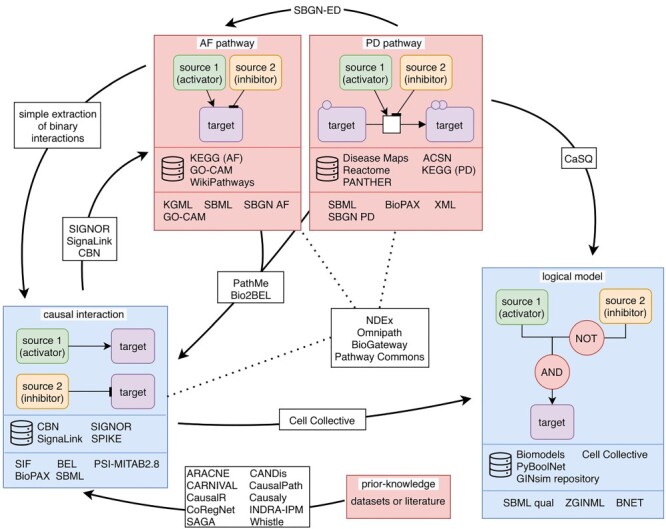
Graphical summary of the manuscript. The figure describes connections between the different types of data resources (causal interaction, AF pathway, PD pathway, logical model and prior knowledge) together with the name of resources that provide this type of information and the possible data formats that can be generated. The directed arrows show possibilities to transform one specific type of data to another type. On each arrow, the tools (or resources) that enable (or provide) the conversion from one resource to another are indicated. For instance, from a PD pathway, it is possible to obtain a logical model using the tool CaSQ. The dotted lines highlight the integrated resources in which several databases have been incorporated.

### Improving the sustainability of resources

The domain of resources available to the logical modeler is a diverse one, ranging from readily available models to useful building blocks that are either directly available from a database or can be extracted via some intermediate software tool. Their continued availability, however, should not be taken for granted, as the existence and maintenance of these resources depends on further development and curation efforts and available grants. The fragility of this knowledge ecosystem is evident, even foundational resources such as KEGG are no exception to this, and it is there that the Elixir initiative provides for a longer term guaranteed availability of carefully curated data [103]. While resources such as Reactome, BioModels and IntAct [104] (a popular and large resource of molecular interactions that is designed to become an important resource for causal molecular information) fall under the umbrella of Elixir, many others do not, and can only find long-term survival if they prepare for integration into these Elixir supported resources. Databases, including SIGNOR and SignaLink, have prepared for this by adopting data representation formats endorsed by the international IMEx consortium, opening the possibility to port their content to IntAct. Other resources such as GO-CAM and NDEx represent new developments that aim to propel component and network curation results to new future applications and are probably safe for some time to be.

## Conclusion

Causal effects between molecular biological entities play an increasingly important role in the analysis and modeling of biological systems. Their relevance for a specific type of analysis often depends on the conditions and context in which they were experimentally observed. Given the extensive variation in metadata describing these conditions and contextual details as found in the large number of resources available online, it is a daunting task for anyone interested in using these causal molecular interactions to find, grasp and understand these differences. This manuscript provides a comprehensive review to help data users appreciate this diversity. We first presented data resources providing causal interactions. Then, we introduced software and pipelines that have been developed to infer causality either from datasets, pathway resources or a combination of both. Finally, we described data formats handling causal information from which data can be downloaded. A summary of these resources, tools and their connection is shown in Figure 3. This set of knowledge constitutes a key element to facilitate the building of logical models to better predict the behavior of a cell system.

Given the range of existing resources, tools and formats, it would serve data users well if the different resources would provide thorough documentation of the curation rules followed to generate the causal statements (e.g. annotations following MI2CAST, additional contextual information, ontologies/controlled vocabularies used). This would give sufficient information to assess if (and how) data are mappable between different resources, thereby improving their interoperability, when possible, by developing tools that would enable to switch between data formats (and resources) without loss of information.

**Key Points**

- Causal molecular interactions are key concepts supporting the assembly of regulatory networks for logical modeling.
- Data resources provide a wide range of causal statements that are commonly manually curated, but tools and algorithms also support the automatic inference of causal statements from biological datasets with implicit causal information.
- Several formats have been developed for the storage of causal statements, with different ranges of contextual information.
- Improved interoperability between resources is dearly needed to facilitate the usability of the available data.

## Supplementary Material

SupplementaryFiles_bbaa390

## Data Availability

No new data were generated or analysed in support of this research.

## Notes

**Vasundra Touré** is a PhD student at the Department of Biology of the Norwegian University of Science and Technology. She is interested in the data management and standardization aspects of causal molecular interactions for both facilitating the curation of causal interaction and the building of logical models.

**Åsmund Flobak** is a Residential Oncologist and an Associate Professor. He focuses on systems medicine research using logic modeling for cancer signaling simulation to predict and understand oncology therapy responses.

**Anna Niarakis** is an Associate Professor at the Department of Biology, Univ Evry, University of Paris-Saclay, affiliated with the laboratory GenHotel in Genopole campus, and a delegate at the Lifeware Group, INRIA Saclay. Her research focuses on the application of computational systems biology approaches in human diseases, including the construction of disease maps, tool development for model inference, network integration and large-scale dynamical modeling.

**Steven Vercruysse** is a Researcher in computer science and computational biology and focuses on building a bridge between human and computer understanding. He created VSM, the general-purpose curation technology that enables the representation of context-rich knowledge, as semantically precise, flexible structures that are intuitive to produce and understand by both humans and computers.

**Martin Kuiper** is a Professor in systems biology at the Department of Biology of the Norwegian University of Science and Technology. His group develops enabling technology for data analysis and knowledge management, among others for the construction of logical models for systems medicine.
