# Supplementary material for: The status of causality in biological databases: data resources and data retrieval possibilities to support logical modeling
Source: Brief Bioinform. 2020 Dec 30;22(4):bbaa390. doi: 10.1093/bib/bbaa390 (PMC8294520; doi:10.1093/bib/bbaa390)
Supplement: SupplementaryFiles_bbaa390 [file supplementaryfiles_bbaa390.pdf]

Supplementary Files

Supplementary File S1: Comparison of the data accession possibilities of the different integrated databases presented in this manuscript.

	Cytoscape plugin	Web service	Libraries	Export
BioGateway [1,2]	BioGateway App	-	-	SIF, all Cytoscape export formats
OmniPath [3,4]	OmniPath App	REST API	Python (pypath), R	SIF, BEL
NDEx [5]	CyNDEx-2 App	REST API	Java, Python, R	SIF, Cytoscape CX
Pathway Commons [6,7]	CyPath2 App	API	Java, Python, R	SIF, TXT, BioPAX L3, JSON-LD, GSEA
PathMe [8]	-	Web application	Python (pathme)	BEL, GraphML, CSV, SIF, Node-link JSON, Cytoscape CX, GSEA
Bio2BEL [9]	-	-	Python (bio2bel)	BEL, GraphML, CSV, SIF, Node-link JSON, Cytoscape CX, GSEA

Supplementary File S2: Some useful links to resources presenting the structure of the different data formats presented in this manuscript.
PSI-MITAB2.8 (GitBook with column definitions):
https://psicquic.github.io/MITAB28Format.html (accessed 10.10.2020)
BEL (official documentation): https://language.bel.bio and
https://biological-expression-language.github.io (accessed 10.10.2020)
GO-CAM (webpage): https://geneontology.cloud (accessed 10.10.2020)
SBMLqual (Level 3 specification webpage):
http://sbml.org/Documents/Specifications/SBML_Level_3/Packages/Qualitative_Models_(qual)
(accessed 10.10.2020)

References

1. Antezana E, Blondé W, Egaña M, et al. BioGateway: a semantic systems biology tool for the life sciences. BMC Bioinformatics 2009; 10:S11
2. Holmås S, Riudavets Puig R, Acencio ML, et al. The Cytoscape BioGateway App: explorative network building from an RDF store. Bioinformatics 2020; 36:1966–1967
3. Türei D, Korcsmáros T, Saez-Rodriguez J. OmniPath: guidelines and gateway for literature-curated signaling pathway resources. Nat. Methods 2016; 13:966–967
4. Ceccarelli F, Turei D, Gabor A, et al. Bringing data from curated pathway resources to Cytoscape with OmniPath. Bioinformatics 2020; 36:2632–2633
5. Pratt D, Chen J, Welker D, et al. NDEx, the Network Data Exchange. Cell Syst. 2015; 1:302–305
6. Cerami EG, Gross BE, Demir E, et al. Pathway Commons, a web resource for biological pathway data. Nucleic Acids Res. 2011; 39:D685–D690
7. Rodchenkov I, Babur O, Luna A, et al. Pathway Commons 2019 Update: integration, analysis and exploration of pathway data. Nucleic Acids Res. 2020; 48:D489–D497
8. Domingo-Fernández D, Mubeen S, Marín-Llaó J, et al. PathMe: merging and exploring mechanistic pathway knowledge. BMC Bioinformatics 2019; 20:243
9. Hoyt CT, Domingo-Fernández D, Mubeen S, et al. Integration of Structured Biological Data Sources using Biological Expression Language. bioRxiv 2019; 631812
